# Case of Inflammation and Obstruction of the Veins, &c.

**Published:** 1831-01-01

**Authors:** 


### XXXV.

### Case of Inflammation and Obstruction of the Veins, accompanied by a swollen State of the Limb. By Mr. T. H. Holberton.

(Med. Chir. Trans. Vol. XVI.)

The present volume of the Medico-chirurgical Transactions so abounds with cases of this kind, that we cannot but perceive the activity of one of the secretaries (Dr. Robert Lee) displayed in the collection. Not that we blame this talented young physician for drawing support for his doctrines from all quarters—but because we cannot help feeling on our guard against the natural endeavour to strengthen an hypothesis by every possible means. The same objection, however, lies against all these cases of phlebitis—namely, that they are different in their nature from the common phlegmatia dolens of lying-in-women. We shall give Mr. Holberton's case, with no abbreviation, as the case is very short.

" *Feb. 26th*, 1830. Robert Willis, æt. 17, No. 3, Providence Court, North Audley Street, has laboured for ten months under pulmonary consumption, in the last stage of which he at present lies, extremely emaciated; with a hurried pulse, hectic fever, copious night sweats, purulent expectoration and diarrhœa, which, *together with pain in the abdomen*, has been very distressing during the last eight weeks.

Three weeks ago he was seized with pain in the right ham, and immediately afterwards *a hot painful swelling of the whole limb*, and a considerable diminution of its muscular power, took place. At present the limb is two-thirds larger than the left, hot and painful. It is colourless, excepting at the groin and inner side of the knee, where the superficial veins are enlarged and distended. The *thigh pits* when rather firmly pressed; is hotter and more painful along the course of the crural vessels than at its outer side; *the femoral vein is clearly traced, like a hard cord, from the groin to where it perforates the tendon of the triceps;* and the saphena at its upper part gives a similar sensation to the touch.

The right foot and ancle are very *œdematous*, and the leg also pits on pressure. There are great heat and tenderness in the hypogastric region, and especially in the right inguinal. Here a greater number of superficial veins are enlarged than on the opposite side, on which also, indeed, some are preternaturally distended. Half a dozen leeches were ordered to the right inguinal region. The wounds bled profusely for some hours, and afforded relief.

27th. The patient is easier in the lower part of the abdomen, and in the right thigh and leg. He can bear pressure rather better along the course of the femoral vessels than yesterday. The *anasarca of the foot is increased.* Being weak and emaciated, he was unwilling to bear a repetition of the leeches, and, as the subsequent treatment consisted only of emollient and anodyne applications, it is unnecessary to enter into detail. He died on Friday morning, 5th inst. (March) 3 o'clock.

*Examination*, on the afternoon of the same day. Present, Dr. Ley, of Half-Moon Street, and Dr. Robert Lee, of Golden Square, the latter of whom was kind enough to visit the patient with me on the 26th ult.

The lungs were found extensively diseased. The veins from the middle of the inferior cava throughout the course of the right femoral, together with the left common iliac, were removed. Externally they were in parts knotty and hard and variously coloured, viz. whitish, dark, greyish, &c. The density of the cellular membrane around each vein corresponded with the quantity of lymph effused into the cavity of the vessel. The arteries in contact with these veins were adherent to them in the same proportion.

*Internal Appearance of the Veins* — In the vena cava for two inches above its bifurcation coagulable lymph, a few drops of puriform reddish matter, and an adventitious membrane, were found; the latter adherent to the inner surface of the vein, which, on the removal of the membrane, presented roughened bloody points. The coats were thickened where they contained the membrane, but healthy above this part.

The right common iliac vein was affected in a similar manner to the inflamed portion of the vena cava, but more intensely, being more loaded with coagulum and having its coats thicker.

The same may be observed of the right external iliac. It contained more adventitious membrane, and its coats were thicker than those of the right common iliac; and the right femoral and its branches, particularly those about the groin, were even, if possible, more distended than the right external iliac.

At the mouth of the right internal iliac, and an inch and a half down the vessel, a firm coagulum and false membrane were found, most materially obstructing, if not entirely preventing, the circulation. Yet this vein did not seem so completely blocked up as those already mentioned, neither were its coats by any means so thick as theirs, though, where the coagulum was lodged, thicker than natural. In cutting off the vessel, about half an inch remained below this point, and was of healthy appearance. The left common iliac and left external iliac were healthy. Serous fluid oozed from the incisions made in the thigh, the cellular membrane of which seemed denser than natural.

The mucous membrane of the lower fifth of the small, and of the whole of the large intestines, was ulcerated. This altered condition of the bowel increased as it descended, and the greatest portion of the lining membrane of the rectum was destroyed."

No one can doubt the existence of phlebitis in the above case—but who can confound it with phlegmatia dolens who has ever had an opportunity of seeing the latter disease?

